# Connected-UNets: a deep learning architecture for breast mass segmentation

**DOI:** 10.1038/s41523-021-00358-x

**Published:** 2021-12-02

**Authors:** Asma Baccouche, Begonya Garcia-Zapirain, Cristian Castillo Olea, Adel S. Elmaghraby

**Affiliations:** 1 Department of Computer Science and Engineering, University of Louisville, Louisville, KY 40292 USA; 2 eVida Research Group, University of Deusto, Bilbao, 4800 Spain

**Keywords:** Breast cancer, Cancer imaging, Cancer screening

## Abstract

Breast cancer analysis implies that radiologists inspect mammograms to detect suspicious breast lesions and identify mass tumors. Artificial intelligence techniques offer automatic systems for breast mass segmentation to assist radiologists in their diagnosis. With the rapid development of deep learning and its application to medical imaging challenges, UNet and its variations is one of the state-of-the-art models for medical image segmentation that showed promising performance on mammography. In this paper, we propose an architecture, called Connected-UNets, which connects two UNets using additional modified skip connections. We integrate Atrous Spatial Pyramid Pooling (ASPP) in the two standard UNets to emphasize the contextual information within the encoder–decoder network architecture. We also apply the proposed architecture on the Attention UNet (AUNet) and the Residual UNet (ResUNet). We evaluated the proposed architectures on two publically available datasets, the Curated Breast Imaging Subset of Digital Database for Screening Mammography (CBIS-DDSM) and INbreast, and additionally on a private dataset. Experiments were also conducted using additional synthetic data using the cycle-consistent Generative Adversarial Network (CycleGAN) model between two unpaired datasets to augment and enhance the images. Qualitative and quantitative results show that the proposed architecture can achieve better automatic mass segmentation with a high Dice score of 89.52%, 95.28%, and 95.88% and Intersection over Union (IoU) score of 80.02%, 91.03%, and 92.27%, respectively, on CBIS-DDSM, INbreast, and the private dataset.

## Introduction

Breast cancer is the most common type of cancer that is leading to death among women, where 41,170 death cases were reported in the United States in 2020 and it represents a rate of 15% of estimated deaths against the other types of cancer^1^. Studies emphasized the importance of frequent mammography screening in order to reduce the mortality rate by detecting the breast tumors early before being spread to normal tissues and other healthy organs^2^. Therefore, mammograms are inspected every day by radiology experts to search for abnormal lesions and detect the location, shape and type of any suspicious regions in the breast. Although this process is considered crucial and requires more precision and accuracy, it remains expensive and exposed to error, due to the increasing number of daily screening mammograms^3^. Medical image segmentation task helps doctors to extract detailed information of the suspicious regions of tumors for further diagnosis and pathology findings. Thus, an automated system can benefit from the high numbers of mammograms and handle this process automatically.

In the last years, the advance in computer vision applications and algorithms has shown remarkable results in developing tools to assist doctors in detecting and segmenting tumors with the lowest possible error in many medical image applications and particularly in mammography^4–6^. Traditional techniques for tumor segmentation, such as region-growing, active contour and watershed, relied on extracting handcrafted features that only represent gray-level, texture, and morphology to label the pixels and indicate the contour surrounding the mass tumors, while excluding the background tissue^7,8^. Computer-aided diagnosis (CAD) development for breast cancer imaging has been recently renewed to cope with the rapid emergence of deep learning algorithms and Artificial intelligence, and it highlights the new systems that may hold real potential to improve clinical care^9–11^.

Recently, the success of deep learning models was highlighted in many medical applications for their capability to extract high-level features directly without knowledge assistance^12–15^. Convolutional neural networks (CNNs) were among the first architectures that attempted to label pixels surrounding objects at different scales and shapes^16^. In respect of medical image segmentation models, the encoder–decoder networks, the fully convolutional network (FCN), were developed and widely known for their ability to extract deep and semantic features and map them with fine-grained details of the target objects over complex backgrounds^17,18^. With the introduction of skip connection, the encoder–decoder architectures were transformed to UNet architecture that was successfully implemented in many medical image segmentation works^19–21^.

Another variation of the FCN, the full resolution convolutional network (FrCN), was introduced by Al-Antari et al.^22^ to segment the detected breast masses, and it produced a Dice score of 92.69% and a Jaccard similarity coefficient of 86.37% on INbreast dataset. Accordingly, Zhu et al.^23^ employed a multi-scale FCN model followed by a conditional random field (CRF) for mammographic mass segmentation, and they achieved Dice score of 90.97% on the INbreast dataset and 91.30% on the DDSM-BCRP dataset. Another work proposed by Singh et al.^24^ was inspired by the FCN architecture and developed a conditional Generative Adversarial Network (cGAN) for breast tumor segmentation. The work achieved a Dice score of 92.11% and an Intersection over Union (IoU) score of 84.55% on the INbreast dataset and a Dice score of 88.12% and an IoU score of 79.87% on a private dataset.

Medical image segmentation usually presents challenging cases; and consequently, FCN network suffered from low segmentation accuracy due to the loss of spatial resolution in the case of small objects and irregular shapes. Therefore, a new model, called UNet, was introduced by Ronneberger et al.^25^ to overcome the limitation of FCN models. UNet proposed to integrate the high-level features from the decoder with the low-level features from the encoder. This fusion was maintained with skip connections that made the UNet architecture adequate in several medical applications and particularly in mammography. A work by Soulami et al.^26^ relied on an end-to-end UNet model for the detection, segmentation, and classification of breast masses in one stage, where the segmentation evaluation showed a Dice score of 90.5% for both DDSM and INbreast datasets. Similarly, Abdelhafiz et al.^27^ implemented a Vanilla UNet model to segment mass lesions in entire mammograms, and it achieved a mean Dice score of 95.1% and a mean IoU score of 90.9% on both digitized film-based and fully digitized MG images.

Inspired by the success of UNet and its variations to improve the overall performance, we propose an architecture that connects two simples UNets, called Connected-UNets. We revisit the original idea of UNet architecture, which added skip connections between an encoder and a decoder network, and we similarly apply another modification of skip connection oppositely between a decoder and an encoder network after cascading a second UNet. Therefore, the final architecture presents two cascaded encoders and decoders that are all alternately connected via different skip connections. We expand the idea of recovering the fine-grained features that are lost in the encoding path of UNet, and we apply it to encode the high-resolution features by connecting them to the previously decoded features. We also add the Atrous Spatial Pyramid Pooling (ASPP) mechanism to the standard UNet architecture and we apply the proposed architecture on two other variations, AUNet and ResUNet, to develop the Connected-AUNets and the Connected-ResUNets. We implement the architectures for segmenting regions of interest (ROI) of breast mass tumors that were previously detected from mammograms of two widely used datasets: Curated Breast Imaging Subset of Digital Database for Screening Mammography (CBIS-DDSM) and INbreast, and from an independent private dataset. We integrate the detection and localization step, presented in a previous work, with the new segmentation step into a final framework that also proposes a preliminary data-enhancement step. In fact, we evaluate the architectures by adding synthetic data generated using an image-to-image translation method, cycle-consistent Generative Adversarial Network (CycleGAN), between the different mammography datasets.

## Results

All experiments using the proposed architecture models were conducted on a PC with the following specifications: Intel(R) Core (TM) i7-8700K processor with 32 GB RAM, 3.70 GHz frequency, and one NVIDIA GeForce GTX 1090 Ti GPU. Python 3.6 was used for conducting all experiments.

### Data preparation

In this segmentation stage, only correctly detected and classified masses by YOLO model were considered and the false predictions were discarded as similarly highlighted in previous works^7,23^. Some cases of mammograms have more than one detected mass lesion, therefore, a total of 1467, 112, and 638 masses were, respectively, considered from the CBIS-DDSM, INbreast, and the private dataset. Our network is applied on single detected ROIs and therefore our intention was to consider mammograms with multiple lesions at the detection stage and treat them separately as single ROIs of mass lesions. The predicted ROI masses were next resized into 256 × 256 using a bi-cubic interpolation in case the original size is small, or using an inter-area resampling interpolation in case it is large. All images were preprocessed to remove additional noise and degradation caused by the scanning technique of digital X-ray mammography^28,29^. Thus, we applied a histogram equalization to enhance the compressed regions and smooth the distribution of the pixels that helps the pixel segmentation. All images were normalized to a range of [0, 1].

To train the proposed segmentation deep learning models, a large amount of annotated samples should be prepared to generalize the learning curve of the models. Due to the limited amount of ROI masses in each dataset, we have augmented the original ROIs four times by rotating them with the angles Δθ = {0°, 90°, 180°, 270°}. We have also transformed them twice differently using the Contrast Limited Adaptive Histogram Equalization (CLAHE) method. Consequently, raw data of single ROI images were augmented six times into a total of 8802, 672, and 3828 of ROI masses were, respectively, prepared from the CBIS-DDSM, INbreast, and the private dataset to train and test the proposed architectures.

### Evaluation metrics and experimental setup

Segmentation stage is evaluated using the Dice similarity score, also called F1-score, which represents a coupled average of the intersection between areas and the total areas as indicated in Eq. (1). Accordingly, we use another evaluation metric, the IoU score, also called the Jaccard score, which is detailed in Eq. (2). A good performance of segmentation is achieved where the pixels surrounding all the masses are correctly segmented and thus a binary mask is generated from the segmented contour of the mass lesions with a high Dice score and IoU score.

1 $${{{\mathrm{Dice}}}}\;{{{\mathrm{score}}}}\left( {A,B} \right) = \frac{{2 \times {{{\mathrm{Area}}}}\;{{{\mathrm{of}}}}\;{{{\mathrm{Intersection}}}}\left( {A,B} \right)}}{{{{{\mathrm{Area}}}}\;{{{\mathrm{of}}}}\left( A \right) + {{{\mathrm{Area}}}}\;{{{\mathrm{of}}}}\left( B \right)}} = \frac{{2 \times \left( {A \cap B} \right)}}{{A + B}}$$

2 $${{{\mathrm{IoU}}}}\;{{{\mathrm{score}}}}\left( {A,B} \right) = \frac{{{{{\mathrm{Area}}}}\;{{{\mathrm{of}}}}\;{{{\mathrm{Intersection}}}}\left( {A,B} \right)}}{{{{{\mathrm{Area}}}}\;{{{\mathrm{of}}}}\;{{{\mathrm{Union}}}}\left( {A,B} \right)}} = \frac{{A \cap B}}{{A \cup B}}$$

To train the proposed architecture models, a learning rate of 0.0001 with Adam optimizer is employed. A weighted sum of Dice and IoU losses is used as a segmentation loss function using the Dice score and IoU score between true and predicted samples, as detailed in Eq. (3).

3 $$\begin{array}{ll}{{{\mathrm{Segmentation}}}}\;{{{\mathrm{loss(true,predicted)}}}}& =\,\, - \left(0.4 \times {{{\mathrm{Dice}}}}\;{{{\mathrm{score}}}}\left( {{{{\mathrm{true,predicted}}}}} \right)\right.\\ &\,\,\quad\left.+\, 0.6 \times {{{\mathrm{IoU}}}}\left( {{{{\mathrm{true,predicted}}}}} \right) \right)\end{array}$$

Each mammography dataset is randomly split into groups of 70%, 20%, and 10%, respectively, for training, testing, and validation sets as shown in Table 1 that highlights the data distribution of each mammography dataset. It is important to highlight that in Table 1 that some of the raw MGs have multiple ROIs. Accordingly, 100 epochs and 8 mini-batches are used to optimize the network parameters with the training and validation sets.

**Table 1:** Data distribution of the mammography datasets.

	Raw MGs data	Raw ROIs data	Augmented data (ROIs*6)	Training data (70%)	Testing data (20%)	Validation data (10%)
CBIS-DDSM	1467	1467	8802	6161	1760	881
INbreast	107	112	672	470	134	68
Private	389	638	3828	2679	766	383

In order to evaluate our integrated framework system, we first define a segmentation accuracy measure to be the mean IoU score for correctly identified ROIs based on a 90% overlap threshold and we refer to it as *IoU_90* score as shown in Eq. (4). Then, a final segmentation accuracy is introduced as an end-to-end accuracy for the two stages, explained in Eq. (5).

4 $${{{\mathrm{IoU}}}}_{90}\;{{{\mathrm{score}}}} = \left\{ {\begin{array}{*{20}{c}} {{{{\mathrm{mean}}}}\left( {{{{\mathrm{IoU}}}}\;{{{\mathrm{scores}}}}\forall {{{\mathrm{ROIs}}}}} \right)} & {{{{\mathrm{if}}}}\;{{{\mathrm{IoU}}}}\;{{{\mathrm{score}}}}\left( {A,B} \right) \ge 90} \\ {{{{\mathrm{Not}}}}\;{{{\mathrm{applicable}}}}} & {{{{\mathrm{if}}}}\;{{{\mathrm{IoU}}}}\;{{{\mathrm{score}}}} < 90} \end{array}} \right.$$

5 $${{{\mathrm{Final}}}}\;{{{\mathrm{segmentation}}}}\;{{{\mathrm{accuracy}}}} = {{{\mathrm{Detection}}}}\;{{{\mathrm{accurcay}}}}\;{{{\mathrm{rate}}}} \times {{{\mathrm{IoU}}}}_{90}\;{{{\mathrm{score}}}}$$

### Quantitative segmentation results

As shown in Table 2, the results are measured for each testing set where we computed the two evaluation metrics for the segmented maps per pixel and compared them to the original ground truth.

**Table 2:** Segmentation performance of our proposed networks on the test sets.

Proposed architectures	Dice score (%)	IoU score (%)	Dice score (%)	IoU score (%)	Dice score (%)	IoU score (%)
	CBIS-DDSM		INbreast		Private	
Standard UNet	78.62	64.87	89.21	79.5	89.87	86.43
Connected-UNets	82.22	69.82	93.36	85.75	95.72	91.95
Standard AUNet	80.39	67.29	91.35	82.59	90.25	88.02
Connected-AUNets	83.84	72.19	93.52	86.01	95.82	92.17
Standard ResUNet	80.94	68.05	92.71	84.58	93.58	89.79
Connected-ResUNets	85.01	73.95	94.13	87.63	95.88	92.27

The comparative results show that the proposed Connected-UNets architecture performs better than the standard UNet in terms of Dice score and IoU score for all the experimental datasets. We also enhanced the segmentation performance of the standard AUNet and ResUNet using the architecture. Accordingly, the results show a comparison of the standard architectures where ResUNet achieved better results than the AUNet, and the later architecture had a better performance than the UNet. The results emphasize the advantages of the attention mechanism and the residual blocks that were added to the simple UNet. We clearly notice an improvement of Dice score by 3.6% using the Connected-UNets, 3.4% using the Connected-AUNets, and 4% using the Connected-ResUNets on the CBIS-DDSM dataset. For the INbreast dataset, we improved the Dice score by 4.15% using the Connected-UNets, 2.17% using the Connected-AUNets, and 1.42% using the Connected-ResUNets. Similarly, we had an improvement of Dice score on the private dataset by 5.85% using the Connected-UNets, 5.57% using the Connected-AUNets, and 2.3% using the Connected-ResUNets.

**Fig. 1:**
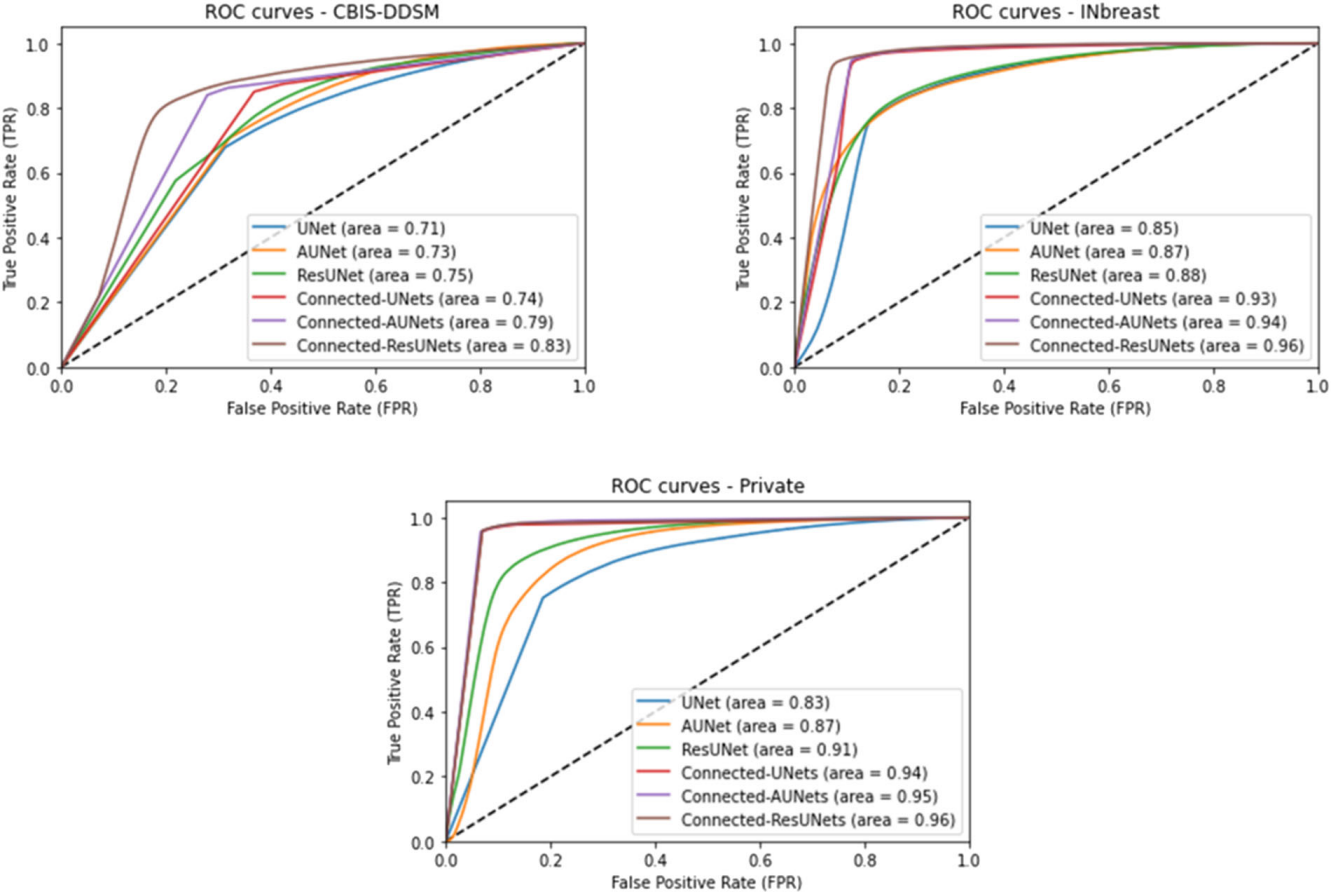
Performance of mass segmentation using the different architectures in terms of ROC curves on the test sets of CBIS-DDSM, INbreast, and the private datasets. ROC curve plots with True positive Rate (TPR) against the False Positive Rate (FPR) and area under curve for pixel-wise evaluation of the standard models (UNet, AUNet, and ResUNet) and for the proposed architecture models (Connected-UNets, Connected-AUNets, and Connected-ResUNets). Subplot on the left shows ROC curve plot for the CBIS-DDSM dataset. Subplot on the right shows ROC curve plot for the INbreast dataset. Subplot on the bottom shows ROC curve plot for the private dataset.

Moreover, the segmentation performance of our proposed Connected-UNets and its variations against the standard UNet, AUNet and ResUNet, was evaluated by the area under curve (AUC) over test sets of all datasets. Segmented images were first generated using each model where pixels were predicted in the range of 0 and 255. After that, predicted images were normalized of scores in the range of 0 and 1. Similarly, ground truth images were normalized to have values of either 0 or 1. Therefore, the problem was transformed into a binary classification task of pixels and consequently receiver operating characteristic (ROC) was computed between the predicted pixels and their true values. Figure 1 shows a comparison of ROC curves where we clearly notice that the proposed architectures outperform all standard models with an average AUC of 0.79 for the CBIS-DDSM, 0.94 for the INbreast, and 0.95 for the private dataset.

**Fig. 2:**
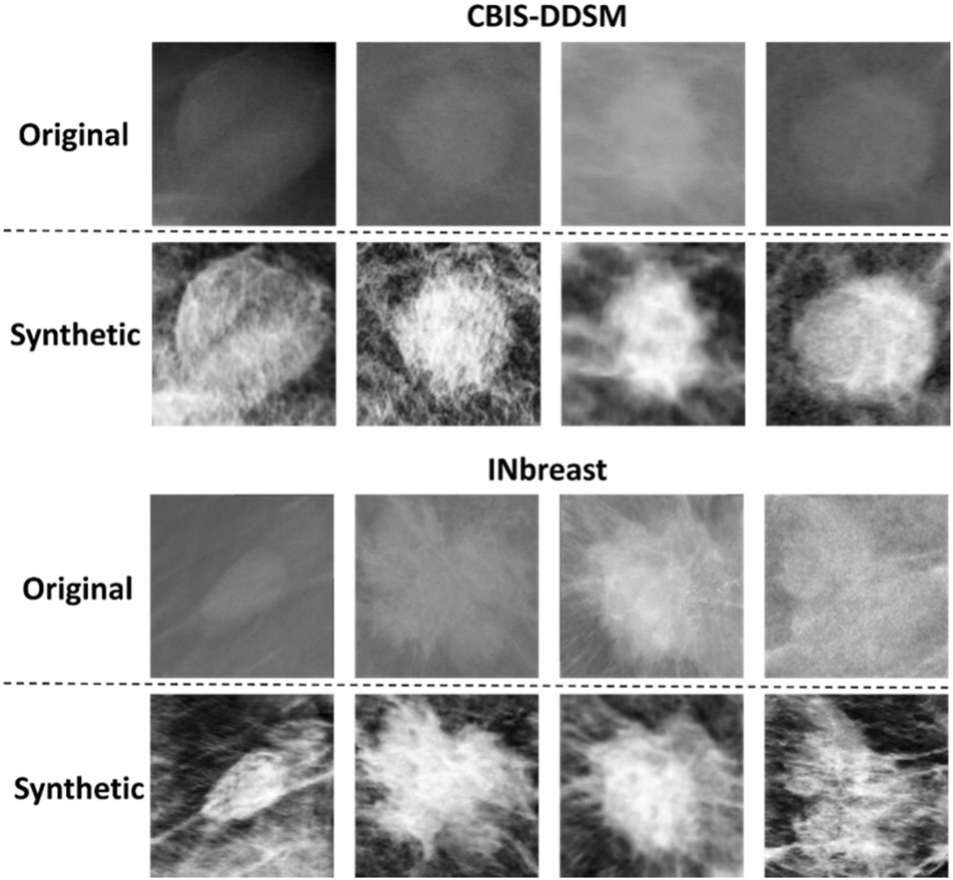
Samples of synthetic data from CBIS-DDSM and INbreast datasets generated by CycleGAN model using the private dataset. Top rows in each subplot show original mammograms respectively from CBIS-DDSM (INbreast) dataset. Bottom rows show their corresponding synthetic mammograms generated by CycleGAN model that was trained respectively between CBISDDSM (INbreast) and the private dataset.

According to Table 2, the private dataset had the best segmentation performance along with the proposed architectures as it represents the best image resolution among the used mammography datasets. Therefore, we applied the CycleGAN model to translate images from CBIS-DDSM and INbreast datasets (i.e., weak domains) into the private dataset (i.e., strong domain). Synthetic images were then created after training the CycleGAN model between the unpaired datasets and generating the new ROI masses for the CBIS-DDSM and the INbreast as shown in the examples below in Fig. 2, where we clearly see the enhanced quality of the new ROI masses that benefit from each dataset’s texture.

Furthermore, we trained the proposed architectures on the original and synthetic images to predict the segmentation mappings. Table 3 shows the improvement of segmentation’s performance of all the standard and architectures using the joined dataset of original and synthetic images. In fact, we notice an increase of Dice score on the CBIS-DDSM by 3.76% using the standard UNet, 3.97% using the standard AUNet, and 4.17% using the ResUNet. Similarly, we have an improved Dice score of 4.8% using the Connected-UNets, 4.11% using the Connected-AUNets, and 4.51% using the Connected-ResUNets.

**Table 3:** Comparison of the proposed architectures after adding synthetic CBIS-DDSM and INbreast.

Proposed architectures	Dice score (%)	IoU score (%)	Dice score (%)	IoU score (%)	Dice score (%)	IoU score (%)	Dice score (%)	IoU score (%)
	CBIS-DDSM without synthetic data		CBIS-DDSM with synthetic data (CycleGAN)		INbreast without synthetic data		INbreast with synthetic data (CycleGAN)	
Standard UNet	78.62	64.87	82.38	72.59	89.21	79.5	93.45	87.54
Connected-UNets	82.22	69.82	87.02	77.07	93.36	85.75	95.16	90.77
Standard AUNet	80.39	67.29	84.36	74.02	91.35	82.59	94.73	89.99
Connected-AUNets	83.84	72.19	87.95	78.89	93.52	86.01	94.89	90.28
Standard ResUNet	80.94	68.05	85.11	76.13	92.71	84.58	94.48	89.59
Connected-ResUNets	85.01	73.95	89.52	80.02	94.13	87.63	95.28	91.03

The integrated framework is finally evaluated using all suggested models. As the end-to-end performance depends on the first detection and localization step which used YOLO model, the segmentation step was first reported using the segmentation accuracy measure IoU_90score that was later multiplied by the detection accuracy rate to form a final segmentation accuracy. Table 4 shows a comparison of final segmentation results of the different models after using the detection accuracy rate of 95.7%, 98.1%, and 98%, respectively, for CBIS-DDSM, INbreast, and the private dataset^30^. Consequently, we reported a final segmentation performance with a maximum accuracy of 86.91%, 93.03%, and 95.39% using the Connected-ResUNets architecture model, respectively, for CBIS-DDSM, INbreast, and the private dataset.

**Table 4:** Final segmentation performance of our proposed networks on the test sets.

Proposed architectures	IoU_90_ score (%)	Final segmentation accuracy (%)	IoU_90_ score (%)	Final segmentation accuracy (%)	IoU_90_ score (%)	Final segmentation accuracy (%)
	CBIS-DDSM		INbreast		Private	
Connected-UNets	90.05	86.18	94.06	92.27	96.99	95.05
Connected-AUNets	90.24	86.36	94.63	92.83	97.22	95.27
Connected-ResUNets	90.82	86.91	94.83	93.03	97.34	95.39

Finally, a comparison of the results of the latest state-of-the-art methods and models to segment the breast masses is listed in Table 5. Our proposed architectures outperformed the UNet model and its current variations. Comparing the Dice score and the IoU score with the other methods shows that we achieved the highest segmentation performance using the architectures on the two public datasets: CBIS-DDSM with a Dice score of 89.52% and an IoU score of 80.02%, and INbreast with a Dice score of 95.28% and an IoU score of 91.03% using the Connected-ResUNets. We surpassed the work of Ravitha Rajalakshmi et al.^31^ by 6.62% Dice score on the CBIS-DDSM dataset, and the work of Li et al.^32^ by 2.56% Dice score on the INbreast dataset.

**Table 5:** Comparison of the proposed architectures and state-of-the-art methods.

Reference	Method	Dataset	Dice score (%)	IoU score (%)
Tsochatzidis et al.^42^	UNet+	CBIS-DDSM	72.2	56.5
Sun et al.^38^	Attention UNet	INbreast	79.1	–
		CBIS-DDSM	81.8	–
Ravitha Rajalakshmi et al.^31^	Deeply supervised U-Net (DS U-Net)	INbreast	79	83.2
		CBIS-DDSM	82.9	–
Dhungel et al.^7^	Deep structured output learning + refinement	INbreast	85	–
Dhungel et al.^12^	CNN + CRF	INbreast	90.06	–
Abdelhafiz et al.^33^	R-UNet	INbreast	90.5	89.1
Zhu et al.^26^	Multi-scale FCN-CRF	INbreast	90.97	–
Wang et al.^40^	ResNet34 + ASPP	INbreast	91.1	–
Singh et al.^27^	conditional GAN (cGAN)	INbreast	91.47	83.58
Al-Antari et al.^25^	Full resolution convolutional network (FrCN)	INbreast	92.63	86.37
Li et al.^32^	Conditional Residual UNet	INbreast	92.72	–
Proposed architectures	Connected-UNets	INbreast	95.16	90.77
		CBIS-DDSM	87.02	77.07
	Connected-AUNets	INbreast	94.89	90.28
		CBIS-DDSM	87.95	78.89
	Connected-ResUNets	INbreast	95.28	91.03
		CBIS-DDSM	89.52	80.02

### Qualitative segmentation results

We applied a post-processing step to all segmented ROI masses by simply removing any outlier point that is far away from the main contour of the lesions. Consequently, we extracted all the possible contours from the binary masks and we only selected the one with the largest area. This was applied for the output of the standard UNet models, the Connected-UNets model and their variations.

**Fig. 3:**
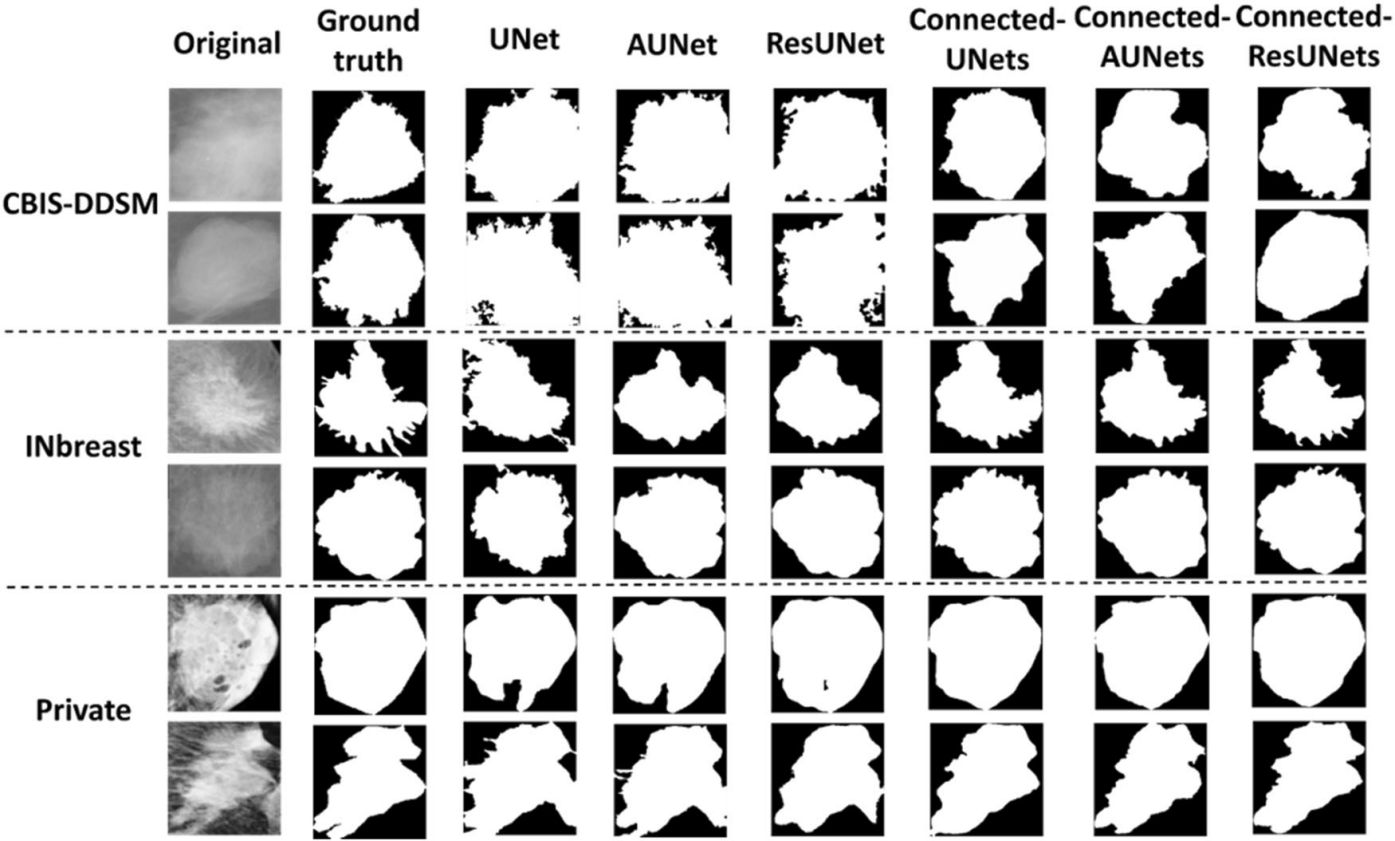
Examples of the segmentation results on the test set of the datasets. Subplot on the top shows two samples of mammograms from the CBIS-DDSM dataset. Subplot on the middle shows two samples of mammograms from the INbreast dataset. Subplot on the bottom shows two samples of mammograms from the private dataset. Each sample from the two rows indicates its corresponding ground-truth (binary) image, segmentation output images of the standard models (UNet, AUNet, and ResUNet) vs segmentation output of the proposed architecture models (Connected-UNets, Connected-AUNets, and Connected ResUNets).

Figure 3 shows examples of the segmented ROI masses generated by the experimental models against their ground truth images. We clearly observe the different quality of the segmentation maps and results of the Connected-UNets and their variations always contain less error and capture more precision compared to the ground truth. Observing the segmentation results, we can see that Connected-ResUNets is more capable to predict the smallest details of the tumor’s boundary than the other architectures. Overall, the proposed architectures outperform the standard architectures and this indicates the power of the proposed architectures to learn complex features through the connections added between the two UNets in the proposed Connected-UNets, which takes advantages of the decoded features as another input in the encoder pathway.

**Fig. 4:**
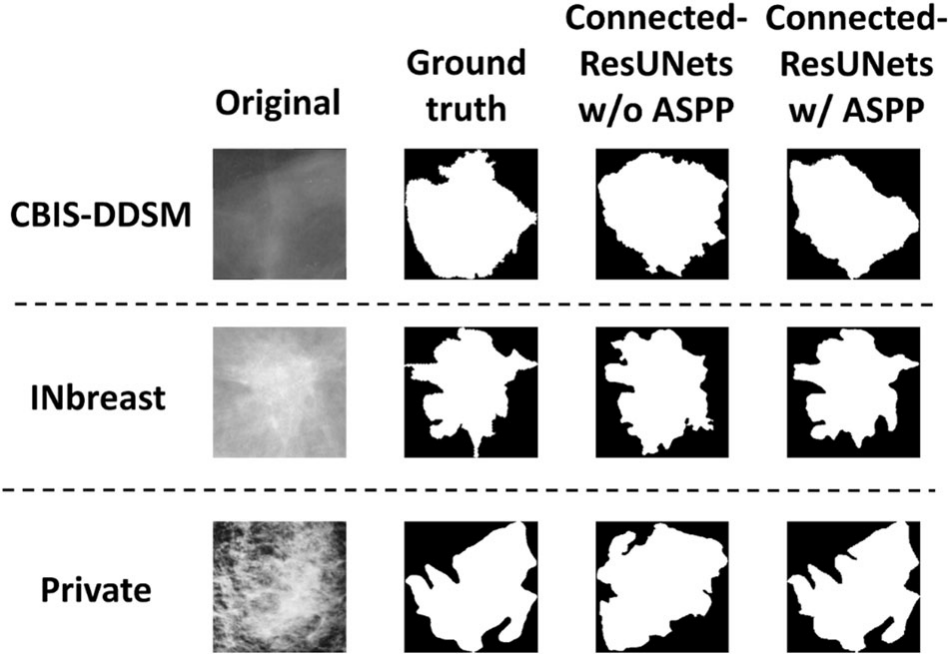
Examples of the segmentation results for the proposed architecture Connected-ResUNets comparing with and without using ASPP block. Top row shows an example of original mammogram from CBIS-DDSM dataset. Middle row shows an example of original mammogram from INbreast dataset. Bottom row shows an example of original mammogram from the private dataset. Each original mammogram indicates its corresponding ground-truth (binary) image, and segmentation output image of the Connected-ResUNets without including the ASPP block vs a segmentation output image of the Connected- ResUNets with including the ASPP block.

Accordingly, a visual comparison of the Connected-ResUNets model, which uses the suggested ASPP block to connect each encoder and decoder, opposed to the same model without using the ASPP block is represented in Fig. 4. We can conclude that the ASPP block added more precision to the segmentation results.

**Fig. 5:**
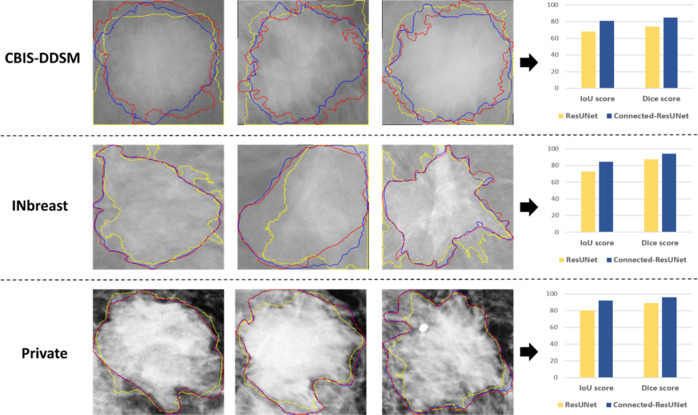
Examples of the segmented masses for the proposed architecture Connected-ResUNets comparing with the ResUNet. Top row shows three examples of mammograms from the CBIS-DDSM dataset. Middle row shows three examples of mammograms from the INbreast dataset. Bottom row shows three examples of mammograms from the private dataset. Each example indicate contours of their ground-truth images, contours of segmentation output images of the standard ResUNet, and contours of segmentation output images of the proposed Connected-ResUNets. Each row also include a comparative bar chart of IoU score and Dice score of ResUNet and Connected-ResUNets models.

After that, a qualitative segmentation comparison of the proposed Connected-ResUNets against the basic ResUNet architecture is presented in Fig. 5. Additionally, a comparison of Dice score and IoU score for each corresponding ROI mass is also achieved. We observe that the proposed architecture model is capable to capture well the smallest details of the tumors having different shapes and sizes from all the used datasets. Hence, it is clear that predicted contours by the Connected-ResUNets is the closest to the ground truth contours and this is also justified with the highest Dice score and IoU score values.

**Fig. 6:**
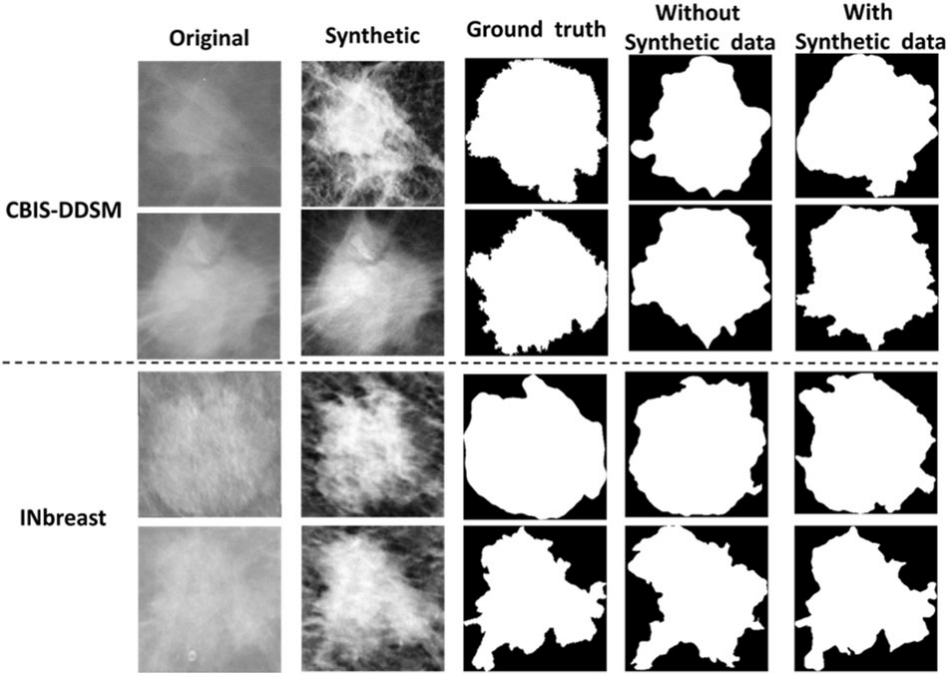
Examples of the segmentation results for the proposed architecture Connected-ResUNets with and without adding the synthetic data. Subplot on the top shows two samples of mammograms from the CBIS-DDSM dataset. Subplot on the bottom shows two samples of mammograms from the INbreast dataset. Each rows indicate original mammogram, its corresponding synthetic mammogram, ground-truth (binary) image, segmentation output image of the Connected-ResUNets model trained without adding synthetic data, and segmentation output image of the Connected-ResUNets model trained with adding synthetic data.

Additionally, we compared the segmentation results of one of the proposed architecture models, Connected-ResUNets, after adding the synthetic images that were generated by the CycleGAN model to the training data. Figure 6 shows a better-segmented contour of the mass tumor using the additional synthetic images. The results of the new training data yield more precise pixel segmentation that is close to the ground truth images. Consequently, the segmentation results’ quality proves the advantage of adding synthetic images to enhance the segmentation quality and it confirms the ability of cross-modality synthesis to augment the size of the data and enhance quality by embracing other similar domains.

**Fig. 7:**
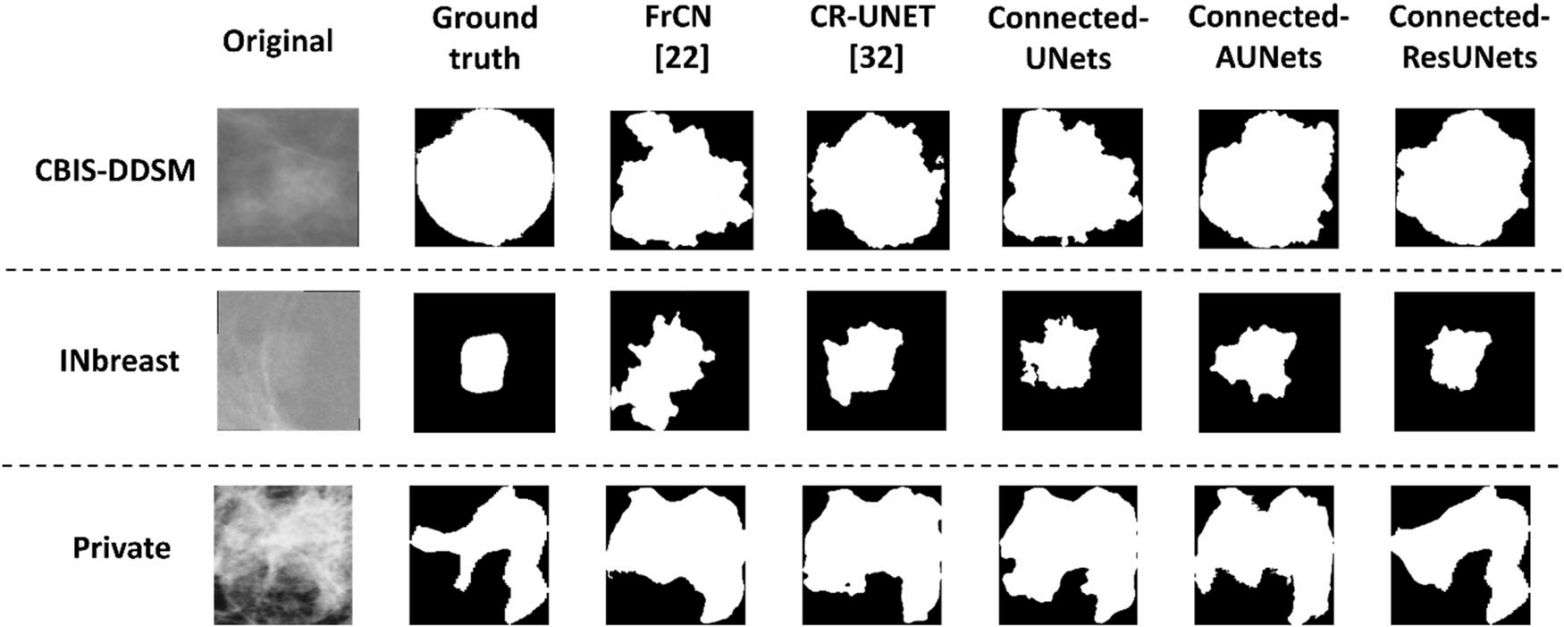
Examples of the segmentation results for the proposed architecture models again two state-of-the-art methods FrCN^22^ and CR-UNET^32^. Top row shows an example of original mammogram from CBIS-DDSM dataset. Middle row shows an example of original mammogram from INbreast dataset. Bottom row shows an example of original mammogram from the private dataset. Each original mammogram indicates its corresponding ground-truth (binary) image, a segmentation output image of a state-of-the-art model FrCN, a segmentation output image of a state-of-the-art model CR-UNET, vs segmentation output image of the proposed architecture models (Connected-UNets, Connected-AUNets, and Connected-ResUNets).

Finally, we applied two state-of-the-art methods that we discussed, by Al-Antari et al.^22^ and Li et al.^32^, to segment ROIs from all the mammography datasets, and visual comparison shows that predictions of two suggested models FrCN and CR-UNET are slightly close to the ground truth images but they do not capture precisely the contours. Examples shown in Fig. 7 are selected to be challenging for segmentation and our proposed architecture models showed better visual results to segment the mass lesions.

## Discussion

Deep learning models have recently achieved remarkable success in segmenting mass tumors in mammograms. Recent studies involved the UNet as one of the state-of-the-art architectures and tried to modify it for a better segmentation performance^22–29^.

In this study, we introduced an architecture, called Connected-UNets, which fully connects two single UNets using additional skip connection paths. The network also employs the ASPP mechanism as a transition block in order to overcome the challenge of losing resolution, particularly in the case of small tumors. The new mass segmentation architecture expands the ability of skip connections to reconstruct the details lost in the encoding pathway by revoking the first decoded features and connect them with the additional encoded inputs. We implemented the architecture on two variations of UNet, as the Attention UNet (AUNet) and the Residual UNet (ResUNet).

The results of the proposed architectures showed the segmentation improvement compared to the basic architectures as shown in Table 2 with a maximum Dice score of 89.52% on the CBIS-DDSM dataset and 95.28% on the INbreast dataset. Moreover, the quantitative evaluation indicated the advantage of the ResUNet and AUNet on segmenting the mass tumors. Hence, the improved architectures’ Connected-AUNets and Connected-ResUNets outperformed the Connected-UNets in all the used mammography datasets. Comparison of the segmentation map results of each model approve the enhancement made to the standard models to provide a precise segmentation of the mass boundaries as shown in Fig. 3.

Limitations of the proposed architectures can occur on the long training time of an average of 0.638 s per epoch, which is due to the high computation of the neural networks that have more trainable parameters than the standard architecture models.

This paper provides an architecture to segment the breast masses in mammograms. The proposed architecture incorporates the recent modifications that were suggested to overcome the challenges of pixel-to-pixel segmentation in medical images, such as attention mechanism, residual blocks, ASPP concept, etc. The improved segmentation performance is made after benefiting from the information decoded using one UNet and propagated again to a second UNet. In addition, synthetic data were created using the CycleGAN model for augmenting the training data. This applies a quality translation between domains in order to embrace the different quality of the existing mammography datasets (i.e., X-ray filter, full-field digital mammography (FFDM)).

In conclusion, this work integrated our recent work^30^ using YOLO model for mass detection and the proposed segmentation architecture models in order to provide a complete clinical tool for mass tumor diagnoses. Future work aims at expanding this tool to assist radiologists for more automatic breast cancer diagnosis such as tumor classification and shape prediction.

## Methods

### Technical background

UNet is one of the state-of-the-art models that was developed for medical image segmentation. Inspired by the FCN. As the name indicates, the network has a symmetric architecture showing a U-shape. It consists of a down-sampling path and an up-sampling path. The remarkable contribution of UNet architecture was the introduction of the skip connections path that added an advantage to the standard architecture. This helps to recover the spatial information that gets lost during the down-sampling path due to the pooling operations.

Many potential scopes of improvement were recently suggested on the UNet architecture to improve its performance and enhance the quality of the segmentation. Ravitha Rajalakshmi et al.^31^ introduced a deeply supervised U-Net model (DS U-Net) associated with dense CRFs to segment suspicious regions on mammograms. The model was tested and gave a Dice score of 82.9% and 79%, respectively, on CBIS-DDSM and INbreast datasets. Accordingly, a Conditional Residual UNet, called CRUNet, was also suggested by Li et al^32^. to improve the performance of the standard UNet for breast mass segmentation, and it achieved a Dice score of 92.72% on the INbreast dataset. Inspired by the residual mechanism, Abdelhafiz et al.^33^ proposed the Residual UNet, called RUNet or ResUNet, by adding residual blocks to the standard convolutional layer in the encoder pathway in order to add deeper effect to the network. The work was applied for mass segmentation and then detected binary maps were fed to a ResNet model for classification into benign or malignant. The segmentation results yielded a Dice score of 90.5% and a mean IoU score of 89.1% on the INbreast dataset. Similarly, Ibtehaz et al.^34^ developed an architecture, called MultiResUNet, which showed a remarkable gain in performance for biomedical image datasets. Another variation of the UNet was suggested using the attention mechanism that showed remarkable success in medical image segmentation^35^. Consequently, Oktay et al.^36^ integrated the attention gate into the standard UNet to propose a new Attention UNet, called AUNet. This improved the prediction performance across CT pancreas segmentation and yielded a Dice score of 83.1%. Similarly, Li et al.^37^ developed an Attention dense UNet for breast mass segmentation that was compared to three basic state-of-the-art models, UNet, AUNet and DenseNet. The suggested model achieved a Dice score of 82.24% on the original DDSM database. In another work suggested by Sun et al.^38^, a attention-guided dense-upsampling network was developed for breast mass segmentation in whole mammograms. The architecture achieved a Dice score of 81.8% on the CBIS-DDSM dataset and 79.1% on the INbreast dataset.

Aligned with the improvement made in encoder–decoder architecture to deal with the limitations encountered in medical images segmentation, the ASPP module was successfully integrated into many networks^39^. This showed effectiveness in breast mass segmentation in a work presented by Wang et al.^40^ that achieved a Dice score of 91.10% and 91.69%, respectively, on the INbreast and DDSM-BCRP datasets.

Studying the UNet architecture showed the unknown architecture’s depth and the restrictive design of skip connections. Therefore, an architecture, named UNet++, was introduced by Zhou et al.^41^ to alleviate the network depth and redesign the standard skip connections. The work was evaluated on six medical images datasets with multiple modalities, and it demonstrated consistent performance for semantic and instance segmentation tasks. A similar variation model, called U-Net+, was employed by Tsochatzidis et al.^42^ to segment ROI mass before integrating it with the classification stage by a CNN model. The segmentation performance showed a Dice score of 0.722 and 0.738, and a Jaccard index of 0.565 and 0.585, respectively, on the CBIS-DDSM and DDSM-400 datasets.

Moreover, to deal with challenging medical images, Jha et al.^43^ presented a DoubleUNets architecture that uses two encoders and two decoders in sequence and an ASPP module. The network showed a better performance than the baselines and UNet on four medical segmentation datasets. In the same context, a Contour-Aware Residual W-Net, called WRC-Net, was suggested by Das et al.^44^, which consists of double UNets. The first UNet was designed to predict objects boundaries and the second UNet generated the segmentation map. Additionally, a variation of the UNet was presented by Tran et al.^45^, named TMD-UNet, which modified the interconnection of the network node, replaced the standard convolutions with dilated convolutions layers, and developed dense skip connections. The network showed superior results than popular models for liver, polyp, skin lesion, spleen, nuclei, and left atrium segmentation.

With the significant attention given to improving the performance of neural networks’ algorithms, many studies have focused on enhancing the quality of medical images that are acquired using multiple imaging modalities. It is often difficult for medical applications to collect enough instances, therefore, synthetic data were recently adopted to increase a size of dataset, within either the same images modality, or using cross-modality translation. Accordingly, Alyafi et al.^46^ employed Deep Convolutional GAN (DCGAN) to generate synthetic mammograms with mass lesions to enhance the classification performance in imbalanced datasets. Another recent technique that was widely used for unpaired image-to-image translation is the CycleGAN, and it was developed by Zhu et al.^47^. This technique learns two mappings by transforming images between two different domains using two GANS and maintains their reconstruction by a cycle-consistency loss and hence the name. In fact, CycleGAN was adopted by Becker et al.^48^ in order to artificially inject or remove suspicious features and thus increase the size of the BCDR and INbreast datasets. Moreover, a cross-modality synthesis approach was introduced by Cai et al.^49^, it was inspired by CycleGAN between CT and magnetic resonance images (MRI) and it was applied on 2D/3D images for segmentation. Another similar work by Hiasa et al.^50^ extended the CycleGAN approach by adding gradient consistency loss and aimed for MRI-to-CT synthesis. The work yielded an improved segmentation accuracy on musculoskeletal images. Upon such an idea, Huo et al.^51^ proposed an end-to-end synthesis and segmentation network (EssNet) to conduct the unpaired MRI-to-CT images synthesis and CT splenomegaly segmentation without using manually annotated CT. This achieved a higher Dice score of 91.88% than the state-of-the-art performance.

### Proposed architecture

**Fig. 8:**
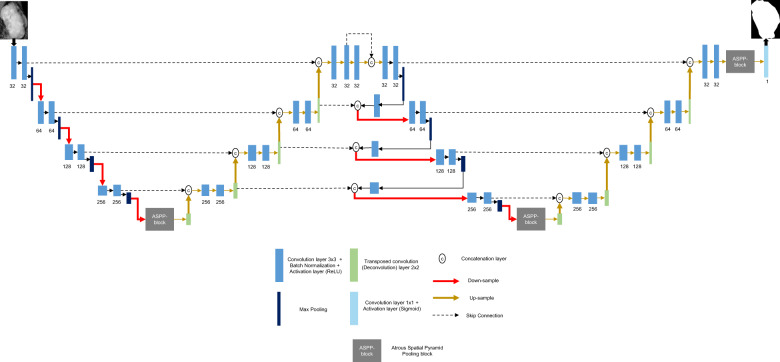
The proposed Connect-UNets architecture. Architecture shows two cascaded encoders (i.e. down-sample pathway) (red arrows) and decoders (i.e. up-sample pathway) (yellow arrows), all alternately connected via skip connection (i.e. dashed lines) and ASPP blocks. An input image is fed to the first block, and a segmentation (binary) image is returned by the last block. Encoders are represented by Convolution layer + Batch Normalization (blue blocks), and Activation layer (dark blue blocks). Decoders are represented by Transposed convolution (green blocks), and Convolution layer + Activation layer (light blue blocks).

Inspired by the efficiency of the skip connections, we propose an architecture, called Connected-UNets, which alternately connects two UNets using additional skip connections. Figure 8 shows an overview of the proposed architecture, where it consists of two standard encoder and decoder blocks and two ASPP blocks for the transition between the two pathways. We suggest connecting the first decoder and the second encoder blocks with additional modified skip connections in order to reconstruct the decoded information in the first UNet before being encoded again in the second UNet. Each encoder block includes two convolution units, which consist of 3 × 3 convolutions followed by an activation ReLU (Rectified Linear Unit) and a batch normalization (BN) layer. A maximum pooling operation is then applied for the output of each encoder block before passing the information to the next encoder. Each decoder block consists of a 2 × 2 transposed convolution unit (i.e., deconvolution layer) that is concatenated with the previous encoder output, and then the result is fed into two convolution blocks, which consist of 3 × 3 convolutions followed by an activation ReLU and a BN layer.

The transition between the down-sample and the up-sample paths is made with an ASPP block. As the name indicates, this technique uses “Atrous” (which means “holes” in French) convolution to allow having a larger receptive field in the transition path without losing resolution.

After going through the first UNet, a second UNet is attached through new skip connections that use information from the first up-sampling pathway. First, the result of the last decoder block is concatenated with the same result after being fed into a 3 × 3 convolution layer followed by an activation ReLU and a BN layer. This serves as the input of the first encoder block of the second UNet. The output of the maximum pooling operations of each three encoder blocks are fed into a 3 × 3 convolutions layers and then concatenated with the output of the last previous decoder block. The result is next down-sampled to the next encoder block. The last encoder block of the second UNet is sent into the ASPP block and the rest is similar to the first UNet, as explained in Supplementary Fig. 1. Finally, the last output is given to a 1 × 1 convolutions layer that is followed by a sigmoid activation layer to generate the predicted mask.

In addition to the proposed architecture that is applied on the standard UNet, we propose another variation by adding an attention block during the up-sampling path, called AUNet model. This integrates the attention mechanism with the skip connections between the encoder and decoder blocks. Indeed, the additional attention block should allow the network to weigh the low-level features (i.e., down-sampled information) before being concatenated with the high-level features (i.e., up-sampled information) during the skip connections. Thus, a new Connected-AUNets architecture is introduced as illustrated in Supplementary Fig. 2.

Motivated by the improvement made to the UNet architecture to be robust enough for segmenting medical images with different scales, we replace the standard convolution blocks with residual convolution blocks, as detailed in Supplementary Fig. 3, to become the Residual UNet (ResUNet), and consequently we proposed a Connected-ResUNets architecture as detailed in Supplementary Fig. 4. Consequently, adding the residual convolution blocks should enhance the UNet architecture to reconcile the features learned at each scale of the down-sampling pathway and take full advantage of the information propagated that may cause degradation of the deep network.

### Image synthesis using CycleGAN

Given our limited size of annotated datasets and differences in their resolutions, we propose to apply image synthesis on our mammography datasets to improve the results of the segmentation. In this study, we propose a cross-domain image synthesis using one of the effective methods: cycle Generative Adversarial Network (CycleGAN)^50^ to enhance our images dataset.

In a CycleGAN architecture, a deep learning model learns the mapping pixel, color distribution, shape and texture between two datasets^52^. In fact, a standard GAN model comprises generator and discriminator networks that are trained alternately such that a generator network tries to produce fake data that is realistic enough to trick the decimator. A CycleGAN is the recent extension of GAN models that is particularly designed for image-to-image translation using unpaired datasets. It has been considered as an effective deep learning technique for style transfer, domain adaptation, and data synthesis^53–55^. The architecture, as shown in Supplementary Fig. 5, consists of two generators and two discriminator networks.

In this work, we developed the CycleGAN model using the available tutorial in Keras webpage (https://keras.io/examples/generative/cyclegan). The generator network consists of nine residual blocks and up-sampling blocks. We did not change the proposed networks and parameters and we prepared our unpaired input datasets to fit with the model.

### Integrated framework: mass detection, image synthesis, and mass segmentation

Our final framework detects and localizes breast masses in a first step, and then segments them in a second step. It also involves an advanced data-enhancement method as a preliminary step before applying the mass segmentation. This step should not only alleviate the low-resolution mammograms, but also augment the size of the mammography datasets.

In fact, the introduced architecture is applied to the ROIs of breast masses that were detected from the previous stage. Our framework applied the You-Only-Look-Once (YOLO) model in previous work^30^ to locate suspicious breast lesions and distinguish between mass and calcification lesions. Therefore, bounding boxes around the suspicious objects were predicted from the entire mammograms. We evaluated the methodology and provided a maximum detection accuracy of 98.1% for mass lesions. Given the different scales of breast masses, our methodology expands some bounding boxes coordinates by adding extra space around the small tumors. Thus, we obtain the ROI images and we scale them to 256 × 256 pixels, which is the optimal input size found experimentally for segmentation networks.

**Fig. 9:**
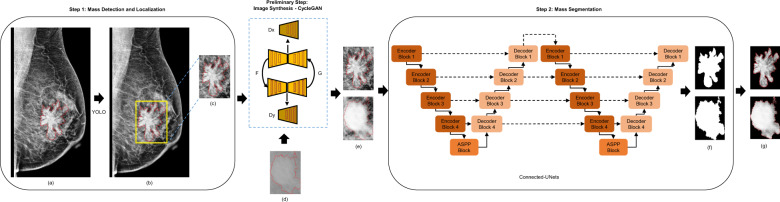
The proposed integrated framework. **a** Original mammogram with ground truth of mass (red). **b** Detected ROI of mass (yellow) superimposed on the original mammogram. **c** Detected ROI (i.e., input mass) obtained with ground truth (red) (Domain X). **d** Detected ROI obtained from a different mammography dataset (Domain Y). **e** Original ROI (Domain X) and Synthetic ROI (transferred from Domain Y to Domain X). **f** Output segmented binary mask of input mass. **g** Segmented output mass where tissue is masked.

Finally, the detected ROI masses’ images and their generated synthetic images are fed directly into the segmentation stage using our proposed architecture as shown in Fig. 9.

### Ethics statement

The clinical data was approved by the institutional review boards and ethical committees of each participation center. The public CBIS-DDSM dataset was registered under clinical trial NCBITAXON and the public INbreast dataset was registered under clinical trial INESC. The private collection was approved by the ethical committee of Mexico under registration INC-2018-1017. Written informed consent was obtained from all patients before enrollment.

### Datasets

In this study, we evaluated the proposed architectures on two public datasets, the CBIS-DDSM and INbreast datasets and a private dataset. CBIS-DDSM^56^ is an updated and standardized version of the Digital Database for Screening Mammography (DDSM) dataset, where images were converted to Digital Imaging and Communications in Medicine (DICOM) format. It contains 2907 mammograms from 1555 unique patients, where 1467 are Mass images. Mammograms were acquired with two different views for each breast (i.e., MLO and CC). Original images have average size of 3000 × 4800 pixels and are associated with their pixel-level ground truth for mass regions. INbreast^57^ is a public database of full-field digital mammography (FFDM) images and prepared in DICOM format. It presents 410 mammograms where only 107 cases include mass lesions in both MLO and CC views of 115 unique patients. The raw images were annotated with experts, and have an average size of 3328 × 4084 pixels.

**Fig. 10:**
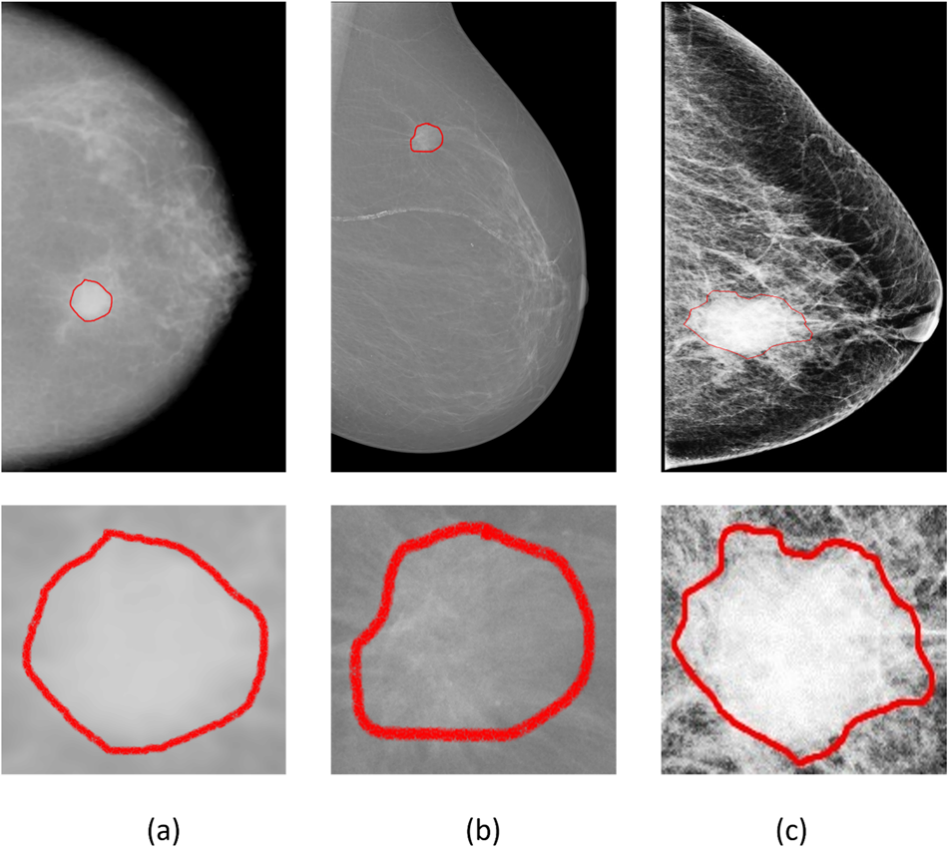
Samples from the public and private mammography datasets with zoomed-in ROI of mass ground truth (red). **a** CBIS-DDSM mammogram example of MLO view. **b** INbreast mammogram example of MLO view. **c** Private mammogram of CC view.

Additionally, the private dataset is a collection of mammograms from the National Institute of Cancerology (INCAN) in Mexico City. The mammograms present stages 3 and 4 of breast cancer of 389 cases with mass lesions obtained from 208 unique patients. Images were collected from CC, MLO, ML, and AT views, and have an average of 300 × 700 pixels. Samples of entire mammograms and their ROI masses are illustrated in Fig. 10. It can be visually observed that the images have different resolutions and this is due to the different modality and tools configurations that were used to acquire and store the mammograms.

### Reporting summary

Further information on research design is available in the Nature Research Reporting Summary linked to this article.

## Supplementary information

Supplementary information.

Reporting summary.

## Data Availability

The public mammography dataset CBIS-DDSM generated and analyzed during the current study is available in the Cancer Imaging Archive, https://wiki.cancerimagingarchive.net/display/Public/CBIS-DDSM. The public mammography dataset INbreast generated and analyzed during the current study is available from the corresponding author Inês Domingues, Porto, Portugal, on resoanble request after signing a transfer agreement. The private mammography dataset generated during and analyzed during the current study is available from the corresponding author Cristian Castillo Olea through the oncologist Dr. Eric Ortiz in the National Institute of Cancerology, Mexico.

## Code availability

The code for custom algorithms and data preprocessing is provided as part of the replication package. It was written in Python v3.6. It is publicly available as a git repository on GitHub at https://github.com/AsmaBaccouche/Connected-Unets-and-more.
