# Supplementary material for: Connected-UNets: a deep learning architecture for breast mass segmentation
Source: NPJ Breast Cancer. 2021 Dec 2;7:151. doi: 10.1038/s41523-021-00358-x (PMC8640011; doi:10.1038/s41523-021-00358-x)
Supplement: Supplementary file 1 — Supplementary information. [file 41523_2021_358_MOESM1_ESM.pdf]

Supplementary Methods

As shown in Supplementary Figure 1, the *ASPP* block is composed of four dilated 3 x 3 convolutions layers with dilation rate $r = \{0, 6, 12, 18\}$ and followed by *BN* layers. The four sup-blocks are next added to create a multi-scaled features block that is finally fed into a 1 x 1 convolutions layer.

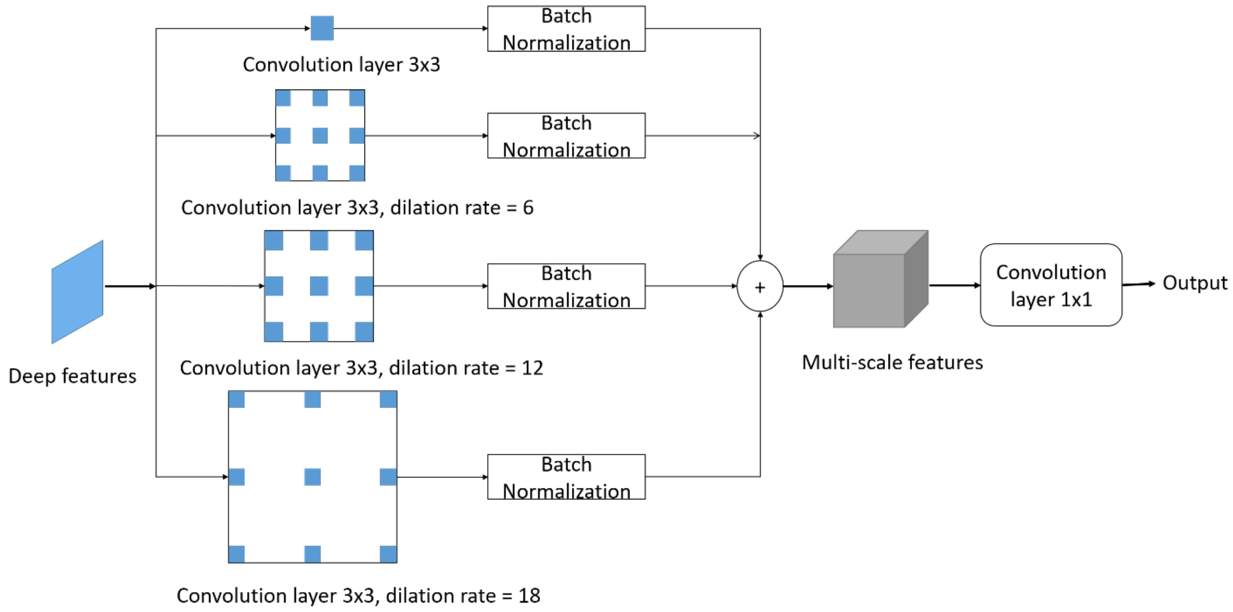

Supplementary Figure 1: The Atrous Spatial Pyramid Pooling (ASPP) block

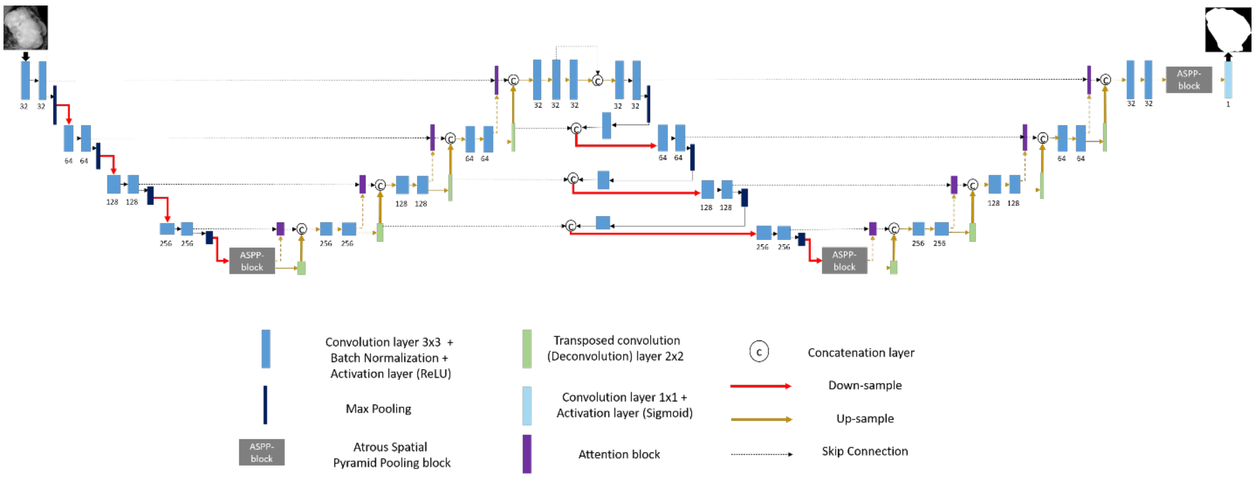

Supplementary Figure 2: The proposed Connect-AUNets architecture

The attention block, as shown in Supplementary Figure3, consists of a 2 x 2 transposed convolutions layer with strides equal to (2,2) and takes low-level features as input. Next, the output is concatenated with the high-level features and the result is fed into a *ReLU* activation layer followed by a 2 x 2 transposed convolutions layer with strides equal to (1,1) and a sigmoid activation layer. This generates the attention map that is next multiplied by the skip connection input to produce the final output of the attention block, which serves as a new input of the decoder block.

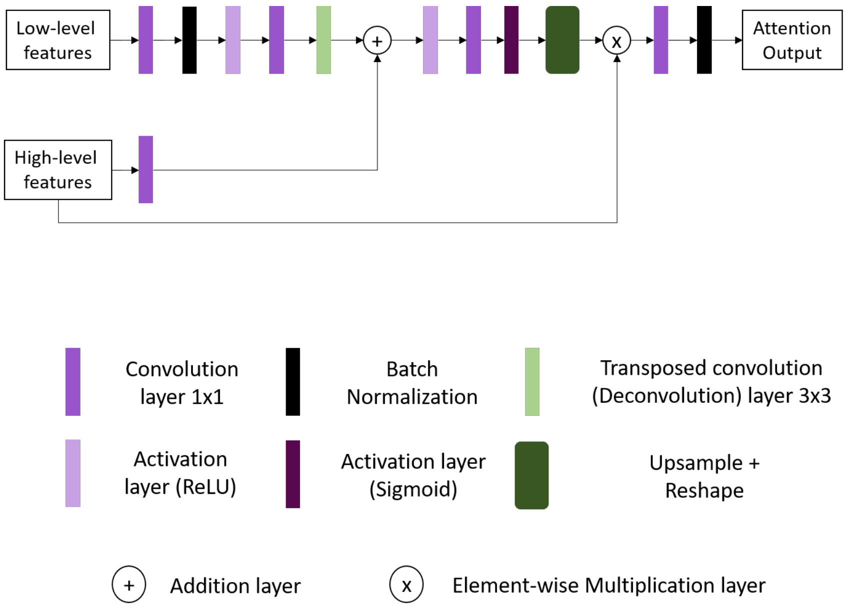

Supplementary Figure 3: The attention block

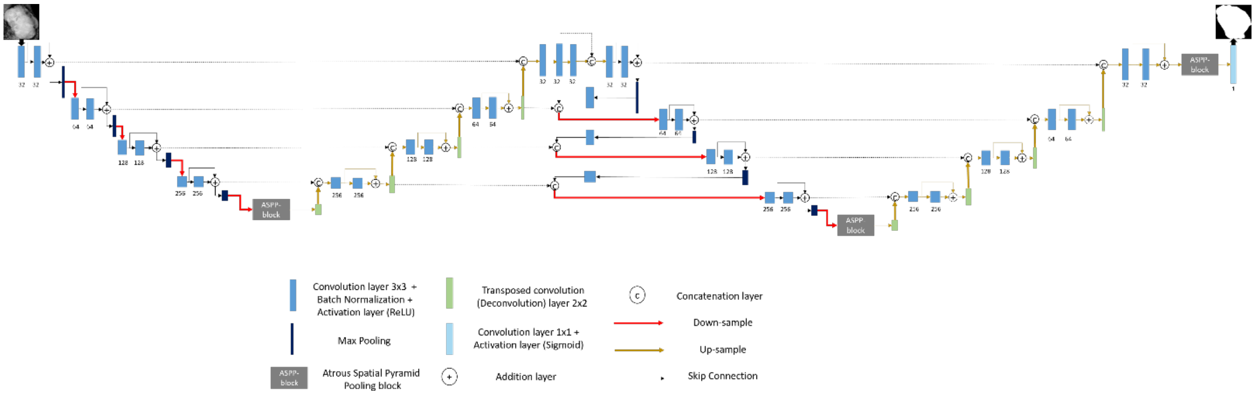

Supplementary Figure 4: The proposed Connect-ResUNets architecture

Given two different domains, where *X* is the source domain (i.e. weak domain) and *Y* is the target domain (i.e. strong domain), the technique applies two generators for cycled image mapping, namely, G: X→Y

and F: Y→X, and two discriminators D_X and D_Y. Following the same logic of GAN, the discriminators are trained to distinguish between the synthetic and the real samples of each domain and thus it minimizes the probabilities p_x and p_Y. However, CycleGAN additionally evokes a cycle consistency for the generators G and F to ensure the reconstruction of the images back to their original domains, where $\mathrm{F(G(X))} = \mathrm{X}$ and $\mathrm{G(F(Y))} = \mathrm{Y}$. This helps the final model to capture the characteristic features of the two domains and transfer the style without requiring any paired dataset. Thus, the network uses the standard adversarial losses and a cycle consistency loss, defined as $\|F(G(X)) - X\|_1 + \|G(F(Y)) - Y\|_1$. We refer to X_{real} as true images coming from domain X and Y_{real} as true images coming from domain Y. However, we refer to X_{fake} as synthetic images translated from domain Y to domain X through generator F, and Y_{fake} as synthetic images translated from domain X to domain Y through generator G.

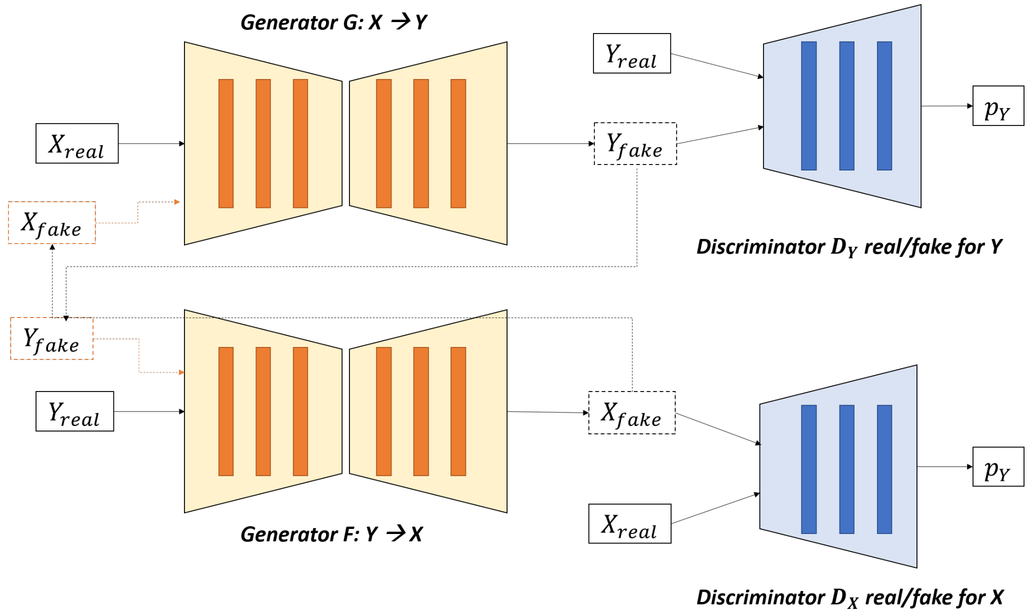

Supplementary Figure 5: CycleGAN architecture for data synthesis
