# Supplementary material for: Connected-UNets: a deep learning architecture for breast mass segmentation
Source: NPJ Breast Cancer. 2021 Dec 2;7:151. doi: 10.1038/s41523-021-00358-x (PMC8640011; doi:10.1038/s41523-021-00358-x)
Supplement: Supplementary file 2 — Reporting summary. [file 41523_2021_358_MOESM2_ESM.pdf]

nature portfolio

Corresponding author(s): Asma Baccouche

Last updated by author(s): Oct 21, 2021

Reporting Summary

Nature Portfolio wishes to improve the reproducibility of the work that we publish. This form provides structure for consistency and transparency in reporting. For further information on Nature Portfolio policies, see our Editorial Policies and the Editorial Policy Checklist.

Statistics

For all statistical analyses, confirm that the following items are present in the figure legend, table legend, main text, or Methods section.

n/a	Confirmed	
☐	☒	The exact sample size (*n*) for each experimental group/condition, given as a discrete number and unit of measurement
☒	☐	A statement on whether measurements were taken from distinct samples or whether the same sample was measured repeatedly
☒	☐	The statistical test(s) used AND whether they are one- or two-sided<br>*Only common tests should be described solely by name; describe more complex techniques in the Methods section.*
☒	☐	A description of all covariates tested
☒	☐	A description of any assumptions or corrections, such as tests of normality and adjustment for multiple comparisons
☒	☐	A full description of the statistical parameters including central tendency (e.g. means) or other basic estimates (e.g. regression coefficient) AND variation (e.g. standard deviation) or associated estimates of uncertainty (e.g. confidence intervals)
☒	☐	For null hypothesis testing, the test statistic (e.g. *F*, *t*, *r*) with confidence intervals, effect sizes, degrees of freedom and *P* value noted<br>*Give P values as exact values whenever suitable.*
☒	☐	For Bayesian analysis, information on the choice of priors and Markov chain Monte Carlo settings
☒	☐	For hierarchical and complex designs, identification of the appropriate level for tests and full reporting of outcomes
☒	☐	Estimates of effect sizes (e.g. Cohen's *d*, Pearson's *r*), indicating how they were calculated

Our web collection on statistics for biologists contains articles on many of the points above.

Software and code

Policy information about availability of computer code

Data collection	All custom algorithms and software used in the collection of data are made available to editors/reviewers in the GitHub repository. Link is listed in the manuscript.
Data analysis	All software used in the analysis of data are publicly available (open source or commercially available). Versions are listed in the Methods and Materials section of the manuscript.<br>The Python computing language was used for analysis and experiments unless otherwise indicated. A statement to this effect is included in the manuscript.

For manuscripts utilizing custom algorithms or software that are central to the research but not yet described in published literature, software must be made available to editors and reviewers. We strongly encourage code deposition in a community repository (e.g. GitHub). See the Nature Portfolio guidelines for submitting code & software for further information.

Data

Policy information about availability of data

All manuscripts must include a data availability statement. This statement should provide the following information, where applicable:

- Accession codes, unique identifiers, or web links for publicly available datasets
- A description of any restrictions on data availability
- For clinical datasets or third party data, please ensure that the statement adheres to our policy

The public mammography dataset CBIS-DDSM generated and analyzed during the current study is available in the Cancer Imaging Archive, https://wiki.cancerimagingarchive.net/display/Public/CBIS-DDSM.
The public mammography dataset INbreast generated and analyzed during the current study is available from the corresponding author Inês Domingues, Porto,

Portugal, on resoanble request after signing a transfer agreement.
The private mammography dataset generated and analyzed during the current study is not publicly available. However, it is available upon reasonable request from the corresponding author Cristian Castillo Olea through the oncologist Dr. Eric Ortiz in the National Institute of Cancerology, Mexico.

Field-specific reporting

Please select the one below that is the best fit for your research. If you are not sure, read the appropriate sections before making your selection.

☒ Life sciences ☐ Behavioural & social sciences ☐ Ecological, evolutionary & environmental sciences

For a reference copy of the document with all sections, see nature.com/documents/nr-reporting-summary-flat.pdf

Life sciences study design

All studies must disclose on these points even when the disclosure is negative.

Sample size	Sample size was determined based on the number of images available in each mammography dataset. Only mammograms with ROI of mass lesions were considered. Images data were randomly split into 70% for training, 20% for testing, and 10% for validation. A maximum of n=8,802 of ROIs were gathered. Sample sizes for all datasets are clearly stated in the manuscript.
Data exclusions	Mammograms with Calcification lesions were excluded from the mammography datasets. Only mammograms with ROI of mass lesions were considered.<br>Three patients have been excluded from the private dataset given their special conditions, that is, multi-focal breast cancer or Paget's disease. One exclusion was due to Paget's disease, that is, a rare form of breast cancer in which cancer cells collect in or around the nipple. The other two were due to multi-focal breast cancer meaning the presence of more than one invasive tumor in one area of their breast.
Replication	All custom algorithms were tested on unseen test sets that were randomly considered, so we conformed that all attempts to replicate experiments were successful.
Randomization	Testing set were selected randomly from each dataset. Images data were randomly split into 70% for training, 20% for testing, and 10% for validation to sample data in the same development stage across samples. We ensured mammograms of the same patient were not included in the same testing sample.
Blinding	Blinding was not applicable in this study for collecting the images datasets from patients. Age, gender, weight and size measurements as the variety of mammograms were known by the individual of data collection. Patients' names were masked as images name only include information about the mammograms as breast position, view and file name.

Reporting for specific materials, systems and methods

We require information from authors about some types of materials, experimental systems and methods used in many studies. Here, indicate whether each material, system or method listed is relevant to your study. If you are not sure if a list item applies to your research, read the appropriate section before selecting a response.

Materials & experimental systems

n/a	Involved in the study	
☒	☐	Antibodies
☒	☐	Eukaryotic cell lines
☒	☐	Palaeontology and archaeology
☒	☐	Animals and other organisms
☐	☒	Human research participants
☐	☒	Clinical data
☒	☐	Dual use research of concern

Methods

n/a	Involved in the study	
☒	☐	ChIP-seq
☒	☐	Flow cytometry
☒	☐	MRI-based neuroimaging

Human research participants

Policy information about studies involving human research participants

Population characteristics	Two public mammography datasets and a private mammography dataset were considered. The public CBIS-DDSM dataset includes 1,555 female patients. The public INbreast dataset includes subpopulation of 115 females with age < 50. The private dataset includes 208 female patients of age between 20-81. All mammogram images show breast, pectoral muscles, nipples, and mass tumor(s).
Recruitment	Participants were recruited with anonymous name. All mammograms were preprocessed to remove artifacts showing personal information. No bias presents in this study.

Ethics oversight	The study protocol was approved by National Center for Biotechnology Information (NCBI); the Breast Research, INESC Port, Portugal; and the National Institute of Cancerology (INCAN), Mexico City, Mexico.

Note that full information on the approval of the study protocol must also be provided in the manuscript.

Clinical data

Policy information about clinical studies

All manuscripts should comply with the ICMJE guidelines for publication of clinical research and a completed CONSORT checklist must be included with all submissions.

Clinical trial registration	1) The CBIS-DDSM dataset: NCBITAXON, http://data.bioontology.org/ontologies/NCBITAXON<br>2) The INbreast dataset: INESC, http://medicalresearch.inescporto.pt/breastresearch/GetINbreastDatabase.html<br>3) The private dataset: Comité de ética México: INC-2018-1017
Study protocol	1) Study protocol for CBIS-DDSM dataset is accessible at:<br>Lee, R. S., Gimenez, F., Hoogi, A. & Rubin, D. L. The Cancer Imaging Archive. http://dx.doi.org/10.7937/K9/TCIA.2016.7O02S9CY (2016). Website: https://wiki.cancerimagingarchive.net/display/Public/CBIS-DDSM<br>2) Study protocol for INbreast dataset is accessible at:<br>Moreira IC, Amaral I, Domingues I, Cardoso A, Cardoso MJ, Cardoso JS. INbreast: toward a full-field digital mammographic database. Acad Radiol. 2012 Feb;19(2):236-48. doi: 10.1016/j.acra.2011.09.014. Epub 2011 Nov 10. PMID: 22078258.<br>The data was provided by Inês Domingues after signing a transfer agreement.<br>3) Study protocol for CBIS-DDSM dataset is accessible at:<br>the National Institute of Cancerology, Mexico by the Oncologist Dr. Eric Ortiz. Website: https://oncologypro.esmo.org/
Data collection	1) The CBIS-DDSM dataset is a curated version of the DDSM dataset introduced in 2017, which is a collection of mammograms released in 1997 from the following sources: Massachusetts General Hospital, Wake Forest University School of Medicine, Sacred Heart Hospital, and Washington University of St Louis School of Medicine. The images are distributed at the full mammography and abnormality level as DICOM files. Full mammography images include both MLO and CC views of the mammograms.<br>2) The INbreast dataset was acquired between April 2008 and July 2010, at a breast center located in a university hospital (Centro Hospitalar de S. Joao, Breast Centre, Porto) with the permission of the Portuguese National Committee of Data Protection and Hospital's Ethics Committee. Annotations were made on OsiriX, an opensource picture archiving and communication system (PACS) workstation.<br>MammoNovation Siemens full-field digital mammography, with a solid-state detector of amorphous selenium was used.<br>3) The private datasetThe data set is made up of stage 3 and stage 4 breast cancer images acquired from National Institute of Cancerology (INCAN), Mexico City, Mexico.
Outcomes	Primary outcome measures for mass lesions segmentation's assessment were predefined using the Dice score and the Intersection-over-Union (IoU) score. The measures define how much the pixels surrounding all the masses (i. e. contours) are correctly segmented comparing to the binary masks. A good performance of segmentation is achieved when the scores have high values (i. e. close to 1). Secondary outcome measures for the segmentation were assessed using an accuracy segmentation that is defined as a composed accuracy between a IoU score more than 90% and a mass lesions detection accuracy.
